# Modeling Human Primary Microcephaly With hiPSC-Derived Brain Organoids Carrying CPAP-E1235V Disease-Associated Mutant Protein

**DOI:** 10.3389/fcell.2022.830432

**Published:** 2022-03-02

**Authors:** Hsiao-Lung An, Hung-Chih Kuo, Tang K. Tang

**Affiliations:** ^1^ Program in Molecular Medicine, National Yang Ming Chiao Tung University and Academia Sinica, Taipei, Taiwan; ^2^ Institute of Biomedical Sciences, Academia Sinica, Taipei, Taiwan; ^3^ Institute of Cellular and Organismic Biology, Academia Sinica, Taipei, Taiwan

**Keywords:** human microcephaly, centriole duplication, cerebral organoid model, ciliopathy, microcephaly gene mutations, CPAP, CENPJ

## Abstract

The centrosome is composed of a pair of centrioles and serves as the major microtubule-organizing center (MTOC) in cells. Centrosome dysfunction has been linked to autosomal recessive primary microcephaly (MCPH), which is a rare human neurodevelopmental disorder characterized by small brain size with intellectual disability. Recently, several mouse models carrying mutated genes encoding centrosomal proteins have been generated to address the genotype–phenotype relationships in MCPH. However, several human-specific features were not observed in the mouse models during brain development. Herein, we generated isogenic hiPSCs carrying the gene encoding centrosomal CPAP-E1235V mutant protein using the CRISPR-Cas9 genome editing system, and examined the phenotypic features of wild-type and mutant hiPSCs and their derived brain organoids. Our results showed that the CPAP-E1235V mutant perturbed the recruitment of several centriolar proteins involved in centriole elongation, including CEP120, CEP295, CENTROBIN, POC5, and POC1B, onto nascent centrioles, resulting in the production of short centrioles but long cilia. Importantly, our wild-type hiPSC-derived brain organoid recapitulated many cellular events seen in the developing human brain, including neuronal differentiation and cortical spatial lamination. Interestingly, hiPSC-CPAP-E1235V-derived brain organoids induced p53-dependent neuronal cell death, resulting in the production of smaller brain organoids that mimic the microcephaly phenotype. Furthermore, we observed that the CPAP-E1235V mutation altered the spindle orientation of neuronal progenitor cells and induced premature neuronal differentiation. In summary, we have shown that the hiPSC-derived brain organoid coupled with CRISPR/Cas9 gene editing technology can recapitulate the centrosome/centriole-associated MCPH pathological features. Possible mechanisms for MCPH with centriole/centrosome dysfunction are discussed.

## Introduction

The centrosome in animal cells is composed of a pair of microtubule-based centrioles surrounded by pericentriolar material (PCM) proteins and serves as the microtubule-organizing center (MTOC) in cells and a regulator of cell-cycle progression (Nigg and Holland, 2018). The centrioles serve not only as the core structure of the centrosome, but also as the basal body: The structure that templates for the formation of cilia and flagellae (Breslow and Holland, 2019). The centrioles are vital for the formation of immotile (primary) cilia and motile cilia, which are essential for cellular signal transduction and locomotion. Centrosome abnormalities trigger mitotic errors and genomic instability, resulting in human disorders such as cancer, while gene mutations that affect the cilia structures and/or ciliary function cause a number of genetic diseases, collectively known as ciliopathies (Reiter and Leroux, 2017).

The primate cerebral cortex has undergone extensive expansion and complexification, as evidenced by the increases in cortical size, surface area, and network density. Much of this expansion can be attributed to the presence of the outer subventricular zone (OSVZ), which represents a separate progenitor layer that contains a unique stem cell subset known as outer radial glia cells (oRG) in the developing cortex of primates/humans (Fietz et al., 2010; Hansen et al., 2010). Autosomal recessive primary microcephaly (MCPH) is a genetically heterogeneous disorder characterized by smaller brain size with mild to severe intellectual disability. Interestingly, many MCPH gene-encoding proteins localize at the centrioles or centrosomes for at least part of the cell cycle, implying the centrosomal role of MCPH proteins in the developing human brain; however, the underlining mechanism is not completely understood. Our team and other teams previously identified six key proteins (STIL, CEP135, CPAP, RTTN, CEP120, and CEP295) that participate in the main process of centriole duplication (Kohlmaier et al., 2009; Schmidt et al., 2009; Tang et al., 2009; Stevens et al., 2010; Tang et al., 2011; Vulprecht et al., 2012; Lin et al., 2013a; Lin et al., 2013b; Comartin et al., 2013; Chang et al., 2016; Chen et al., 2017; Tsai et al., 2019). Among these, mutations in the *STIL*, *CEP135*, *CPAP*, and *RTTN* genes were reported to cause MCPH in humans (Thornton and Woods, 2009; Jayaraman et al., 2018; Vandervore et al., 2019).

Recent studies from many labs including ours linked several abnormal features of neurogenesis in developing brains to defective *CPAP/CENPJ* gene using mouse models (McIntyre et al., 2012; Insolera et al., 2014; Lin et al., 2020). However, unlike primates, the mouse cortex possesses only a few oRG cells (Wang et al., 2011). Thus, the current knowledge obtained from microcephalic mouse models may not directly apply to humans. Recently, the hiPSC-derived 3D organoid culture system was developed, which recapitulates human brain development *in vitro* (Lancaster et al., 2013; Ostrem et al., 2017; Renner et al., 2017). Moreover, several hiPSC-derived brain organoids carrying MCPH-associated genes have recently been established (Lancaster et al., 2013; Gabriel et al., 2016; Li et al., 2017; Zhang et al., 2019; Gabriel et al., 2020). However, the information obtained from these studies is still limited.

In this study, using the CRISPR/Cas9 genome editing system, we introduced a homozygous *CPAP/CENPJ* missense mutation (c.3704A > T; E1235V) previously identified in MCPH patients (Bond et al., 2005) into an isogenic human-induced pluripotent stem cell (hiPSC) line. We examined the cellular and phenotypic features of wild-type and mutant hiPSCs and their derived brain organoids. Our results showed that hiPSCs carrying the CPAP-E1235V mutant produced short centrioles but long cilia. Interestingly, the CPAP-E1235V mutation induced p53-dependent neuronal cell death, resulting in the production of smaller brain organoids. Furthermore, the CPAP-E1235V mutation induced NPC spindle misorientation and caused premature neuronal differentiation. Herein, we provide new insights into the cellular pathogenesis of how a naturally occurring CPAP-E1235V mutation may cause primary microcephaly in humans.

## Materials and Methods

### Human-Induced Pluripotent Stem Cell (hiPSC) Culture

hiPSC (GM25256*C) was purchased from the Coriell Institute for Medical Research, United States. The hiPSCs were cultured in medium containing DMEM/F12 (Gibco, cat. no. 11330032), 20% knockout serum replacement (KSR, Gibco, cat. no. 10828028), 1 mM GlutaMAX (Gibco, cat. no. 35050061), 0.1 mM MEM non-essential amino acid (Gibco, cat. no. 11140050), 0.1 mM beta-mercaptoethanol (Sigma, M6250), and 20 ng/ml recombinant human fibroblast growth factor-basic/FGF-2 (Peprotech, cat. AF-100-18B). The culture medium was replaced every day. *Mycoplasma* was verified as being contamination free routinely every month with the PCR method using an EZ-PCR *Mycoplasma* Detection Kit (cat. no. 20-700-20, Biological Industries). hiPSCs were passaged every week using 0.1% (wt/vol) Dispase Ⅱ (Sigma, D4693) for 20 min. hiPSC colonies were then detached from the culture dish, disassociated into small pieces by pipetting gently, and aggregates were seeded onto Matrigel (Corning, cat. no. 354230)-coated plates.

### Genome Editing

The isogenic hiPSC lines carrying the homozygous c.3704A>T mutation in the *CPAP/CENPJ* gene were generated using the CRISPR/Cas9 genome editing system. For genome editing, the target guide-RNAs (gRNAs) were designed using the online software CHOPCHOP, which were cloned into the PX458 vector (Addgene, plasmid #48138) according to the protocol described by Ran et al. (2013). The gRNAs targeting sequences used for generating the c.3704A > T homozygosity point mutation in exon 16 of human *CPAP* were as follows: F: 5′-ACA​GAA​AAA​CAT​TAC​CCA​GA-3′ and R: 5′-TCT​GGG​TAA​TGT​TTT​TCT​GTC -3′. For delivering the gene editing constructs into hiPSCs, we co-transfected 1 μg gRNA with homology-directed repair (HDR) template (100bp single-strain DNA, the final concentration was 10 nM) into hiPSCs by tube electroporation (Lonza Nucleofector) according to the manufacturer’s instructions. Meanwhile, the cells were treated with small molecules, RS-1 (7.5 μM, Sigma, R9782) and SCR7 (5 μM, Sigma, SML1546), to increase HDR until cell sorting. GFP was used as a marker with which to indicate successful delivery, and GFP-positive cells were collected using the cell sorter (BD, FACSJazz) within 48 h of electroporation. The GFP-positive cells were seeded onto Matrigel-coated culture dishes and treated with RS-1 (7.5 μM) and SCR7 (5 μM) for an additional 48 h. hiPSC colonies were picked and expanded. Genomic DNAs and total RNAs were isolated from each hiPSC colony and PCR and rtPCR analysis were performed using the following primers: F: 5′-ATG​TTC​CTG​ATG​CCA​ACC​TCT-3′, R: 5′-TCA​CAG​CTC​CGT​GTC​CAT​TAG-3′ for CPAP-full length cDNA; F: 5′-AAC​TTG​CTC​CAC​CCT​CTC​ATT​A-3′ and R: 5′-CCT​TCT​GAG​CAC​AGA​TGA​ACA​G-3′ for *CPAP* exon 16. PCR products were subjected to DNA sequencing to confirm the mutation sequences.

### Western Blot Analysis

Cell lysates were prepared from CPAP wild-type and mutant cells using the RIPA buffer with proteinase inhibitors containing aprotinin (1 mg/ml), pepstatin (1 mg/ml), and leupeptin (1 mg/ml), as previously described (Tang et al., 2011). The protein lysates were separated in sodium dodecyl sulfate polyacrylamide gel electrophoresis (SDS–PAGE) and incubated with the appropriate antibodies.

### TUNEL Assay

For the cell apoptosis assay, cells were fixed using 4% PFA at room temperature (RT) for 20 min and blocked with 4% normal goat serum containing 0.25% Triton-X 100 in PBS at RT for 1 h. The fixed cells were washed in PBS and the fragmented DNA was labelled using the terminal deoxynucleotidyl transferase-mediated dUTP nick end labeling (TUNEL) kit (Roche 11684795910), according to the manufacturer’s instructions.

### Cilium Induction

For the cilium assembly experiment, hiPSCs were incubated in the culture medium, containing DMEM/F12 medium without FGF2, 0.5% KSR, 1 mM GlutaMAX, 0.1 mM MEM non-essential amino acid, and 0.1 mM beta-mercaptoethanol for 48 h. Cells were fixed in cold-methanol (100%) for 5 min at −20°C and immunostained with the appropriate antibodies against ciliary proteins.

### Spindle Orientation Analysis

The brain organoid cryosections were labeled with antibodies against p-VIMENTIN and PERICENTRIN to visualize the mitotic NPCs and centrosomes, respectively. The DNA was counterstained with DAPI. Images were obtained using a confocal microscope (Zeiss LSM 700 stage; Carl Zeiss) and analyzed with the ImageJ software (NIH) or ZEN software (Zeiss). The spindle angle (θ) of mitotic NPCs at the anaphase was calculated by drawing a line connecting the two centrosomes (spindle axis) and a line parallel to the apical surface using the ImageJ software.

### Generation of Human-Induced Pluripotent Stem Cell-Derived Brain Organoids

hiPSC-derived brain organoids were generated according to a published protocol (Lancaster and Knoblich, 2014). In brief, hiPSC colonies were treated with accutase (Gibco, cat. no. A1110501) to dissociate the colonies into single cells. For embryoid body (EB) formation, around 9000 single hiPSC cells were seeded onto each well of the round-bottomed ultra-low attachment 96-well plate (Corning, cat. no. 7007), and cultured in EB medium containing DMEM/F12, 20% knockout serum replacement, 1 mM GlutaMAX, 0.1 mM MEM non-essential amino acid, 0.1 mM beta-mercaptoethanol (Sigma, M6250), 4 ng/ml FGF-2, and 10 μM Y-27632 (ROCK inhibitor, Selleck. cat. no. S1049). On Day 3, the EBs were cultured in EB medium without FGF-2 and Y-27632. On Day 6, the EBs were transferred onto the well of a flat-bottomed ultra-low attachment 24-well plate (Corning, cat. no. 3473) for neural induction (NI). The NI medium consisted of DMEM/F12, 1% N-2 supplement (Gibco, cat. no. 17502048), 1% GlutaMAX, 1% MEM non-essential amino acid, and 1 μg/ml heparin (Sigma, H3149). On Day 12, the EBs were embedded in Matrigel growth-factor-reduced basement membrane matrix (Corning, cat. no. 354230) and cultured in differentiation medium without vitamin A, which contained 50% DMEM/F12, 50% Neurobasal medium (Gibco, cat. no. 21103049), 0.5% N-2 supplement, 1% B27 without vitamin A (Gibco, cat. no. 12587010), 1% GlutaMAX, 0.5% MEM non-essential amino acid, 0.025% human insulin, and 0.035% beta-mercaptoethanol (Sigma, M6250). On Day 16, Matrigel-embedded organoids were transferred into a spinning bioreactor (Corning, glass spinner flask, 4500125. Chemglass life Sciences, Stir plate, CLS410009) and cultured in differentiation medium containing 50% DMEM/F12, 50% Neurobasal medium, 0.5% N-2 supplement, 1% B27 containing vitamin A (Gibco, cat. no. 17504044), 1% GlutaMAX, 0.5% MEM non-essential amino acid, 0.025% human insulin (Sigma, I9278), and 0.035% beta-mercaptoethanol (Sigma, M6250). Agitation speed was set at 25 rpm and the medium was changed every 7 days. The experiments involving hiPSC-derived brain organoids were approved by the IRB of Academia Sinica.

### Antibodies

For immunostaining, primary and secondary antibodies were diluted in 1X PBS (pH 7.4) with 10% normal goat serum. The primary antibodies used in this study included rabbit anti-CPAP [1:400, (Hung et al., 2000)], rabbit anti-CENTRIN-2 [1:800, (Tang et al., 2009)], rabbit anti-CEP120 [1:1000, (Lin et al., 2013b)], rabbit anti-CEP295 [1:800, (Chang et al., 2016)], rabbit anti-CENTROBIN [1:1000, (Tang et al., 2009)], rabbit anti-NANOG (1:200, Cell Signaling 4903), mouse anti-OCT-4 (1:200, Cell Signaling 75463), mouse anti-PAX6 (1:200, BD Biosciences 561462), rabbit anti-PAX6 (1:200, BioLegend 901301), mouse anti-p53 (1:100, Santa Cruz Biotechnology sc-126), mouse anti-REELIN (1:200, MBL D223-3), mouse anti-phosphorylated VIMENTIN (1:400, MBL D076-3), mouse anti-SATB2 (1:200, Abcam ab51502), rabbit anti-TBR1 (1:200, Abcam ab31940), mouse anti-TUJ1 (1:800, Covance MMS-435P), rabbit anti-Ki67 (1:600, Abcam ab16667), mouse anti-ZO-1 (1:200, Invitrogen 339100), mouse anti-CENTRIN-3 (1:800, Abnova H0000170-M01), mouse anti-Acetylated TUBULIN (1:600, Sigma-Aldrich T6793), rabbit anti-CEP162 (1:1000, Abnova PAB22408), rabbit anti-ARL13B (1:800, Proteintech 17711-1-AP), rabbit anti-STIL (1:200, Bethyl A302-442A), rabbit anti-POC5 (1:200, Bethyl Laboratories A303-341), rabbit anti-POC1B (1:200, Thermo Fisher PA5-24495), rabbit anti-CEP164 (1:500, Novus Biologicals NBP1-81445), rabbit anti-cleaved CASPASE3 (1:200, Cell Signaling 9664), and rabbit anti-PERICENTRIN (1:1000, Abcam ab4448). Secondary antibodies were AlexaFluor 488-, 568-, 647-conjugated goat anti-rabbit (Life Technologies A-11034, A-11036, A-21245) or goat anti-mouse (Life Technologies A-11029, A11031, A-21236) IgG. All secondary antibodies were used at a 1:400 dilution. For western blotting, the primary and secondary antibodies were diluted in PBST (0.1% Tween-20) with 5% Difco Skim Milk (BD 232100). The primary antibodies used in this experiment were rabbit anti-CPAP [1:1000, (Hung et al., 2000)] and mouse anti-*α*-TUBULIN (1:10000, Sigma-Aldrich T9026). The secondary antibodies were goat anti-mouse IgG labeled HRP (1:2000, PerkinElmer NEF822) or goat anti-rabbit IgG labeled HRP (1:3000, PerkinElmer NEF812).

### Confocal Microscopy

Brain organoids were fixed in 4% paraformaldehyde (PFA) at 4°C for 30 min, washed in PBS three times, dehydrated in 30% sucrose overnight, embedded in gelatin (7.5%)/sucrose (10%), and then cryosectioned at 25 μm for imaging. For immunofluorescence imaging, cells were fixed either in methanol at −20°C for 5 min or in 4% PFA at RT for 20 min, and then blocked with 10% normal goat serum containing 0.25% Triton-X100 in PBS at RT for 30 min. The cryosections were blocked and permeabilized with 4% normal goat serum containing 0.25% Triton-X 100 in PBS at RT (humidified chamber) for 1 h. Cells or cryosections were incubated with the indicated primary antibodies at 4°C overnight, washed in PBS three times, and then incubated with conjugated-secondary antibodies (Alexa Fluor 488, 568 and 647) at 4°C overnight. Samples were then mounted in mounting medium containing DAPI (Abcam, ab104139). The images of cells or cryosections were obtained using an LSM 700-stage laser scanning confocal microscope (Zeiss). To measure the centriole length in the G2-phase cells, super-resolution images were taken using the LSM 880 with an Airyscan confocal microscope (Zeiss). Images were analyzed using the ZEN software (Zeiss).

### Statistics

Statistical analysis was performed using GraphPad Prism 9 software or Excel. Data are presented as the mean ± standard error of the mean (SEM). Significant differences were analyzed using the two-tailed unpaired Student’s *t*-test. n.s: not significant; *p < 0.05; **p < 0.01; ***p < 0.001.

## Results

### Generation and Characterization of Human-Induced Pluripotent Stem Cell Expressing the CPAP-E1235V Mutant Protein

**FIGURE 1:**
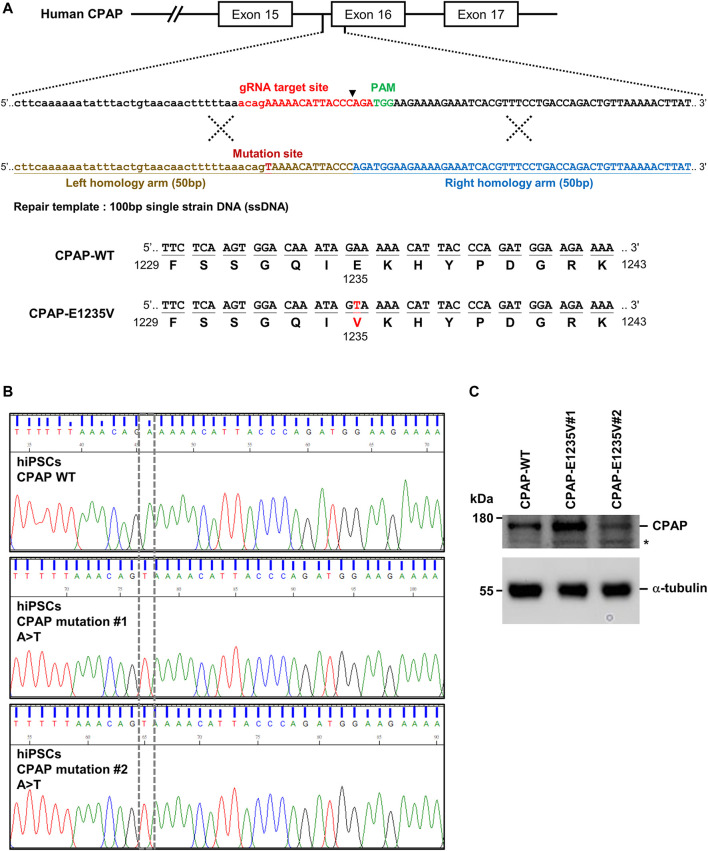
Generation of CRISPR/Cas9-mediated homozygous point mutation in the *CPAP/CENPJ* gene. **(A)** The scheme of homologous recombination between endogenous *CPAP* wild-type (WT) and exogenous repair template (single strain DNA, ssDNA) harbored A>T point mutation. Black inverted triangle: Cas9-cutting site. **(B)** Sanger sequencing results of two independent mutant clones displaying homozygous A>T point mutation in exon 16 of *CPAP*, which resulted in Glutamic acid (E) being replaced by Valine (V) at amino acid 1235. **(C)** CPAP protein expression level in WT and two independent mutant hiPSC clones. Black star symbol indicated non-specific bands.

A missense mutation 3704A-T (c.3704A>T), resulting in the amino acid change E1235V (p.Glu1235Val) in the *CPAP/CENPJ* gene, was previously reported to cause MCPH (Bond et al., 2005). To generate the *CPAP* c.3704A>T mutation carrying the E1235V mutant protein, human iPSCs (GM25256*C), originally purchased from the Coriell Institute for Medical Research, were used to produce the isogenic mutant clones using the CRISPR/Cas9 genome editing system (Ran et al., 2013) (Figure 1A). Two independent mutant hiPSC clones (CPAP-E1235V#1 and #2) were obtained and RT-PCR analysis of the full-length *CPAP* cDNA confirmed a homozygous mutation at 3704 A→T in exon 16 of *CPAP*, leading to an E1235V amino acid substitution (Figures 1A,B); no other variants were present in the *CPAP* gene of mutant iPSCs. The original human iPSC clone without the CRISPR/Cas9 treatment is regarded as hiPSC-CPAP-WT. A further western blot analysis showed that the protein size and expression level of CPAP (WT and CPAP-E1235V mutants) revealed no significant difference between hiPSC-CPAP-WT and hiPSC-CPAP-E1235V mutant clones (Figure 1C), suggesting that the generated *CPAP* 3704 A→T point mutation did not appear to alter CPAP and its protein expression level.

**FIGURE 2:**
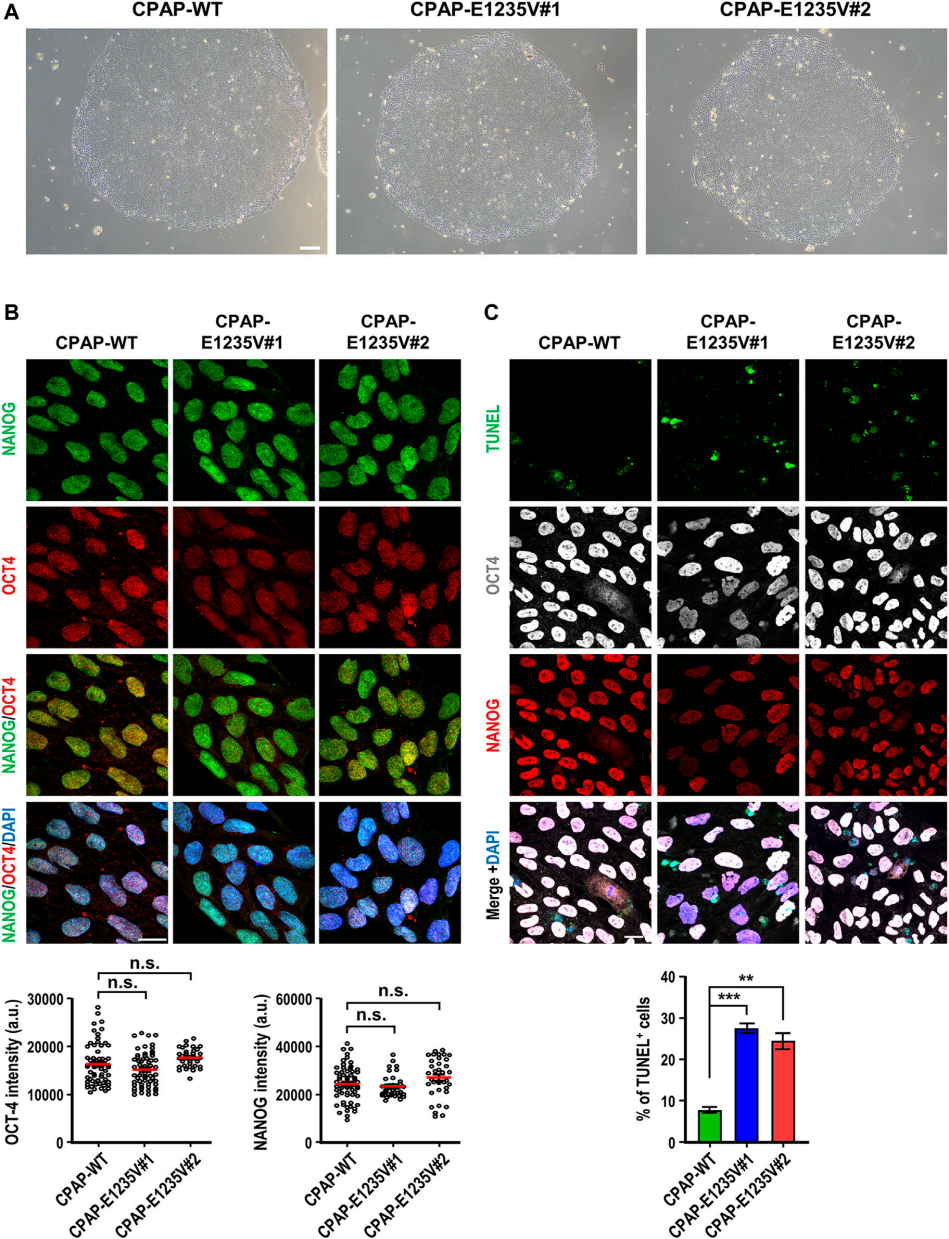
Characterization of CPAP-E1235V mutant hiPSC clones. **(A)** Bright field colony morphology of CPAP-WT and two mutant cell clones. **(B)** Immunofluorescent staining and quantification for pluripotency markers: NANOG (green), OCT4 (red), and DNA (DPAI, blue). *n = 64* for CPAP-WT; *n = 37* for CPAP-E1235V#1; *n = 36* for CPAP-E1235V#2. Data represent mean ±SEM. **(C)** Staining and quantified results of apoptosis marker TUNEL in WT and mutant hiPSC clones. *n = 664* for CPAP-WT; *n = 281* for CPAP-E1235V#1; *n = 619* for CPAP-E1235V#2. Data are presented as the mean ±SEM from a pool of n cells from three independent experiments. n.s.: not significant; **p < 0.01; ***p < 0.001. Scale bar: 200 μm in **(A)**, 20 μm in **(B,C)**.

Importantly, all hiPSC-CPAP-E1235V mutant clones revealed a similar cell morphology (Figure 2A) and expressed pluripotent stem cell markers (NANOG and OCT4) with a similar intensity as compared to their isogenic hiPSC-CPAP-WT control clones (Figure 2B), suggesting that the CPAP-E1235V mutation did not alter the pluripotency properties. A further analysis by TUNEL staining revealed a significant increase in apoptosis of the pluripotent stem cells (NANOG^+^ and OCT4^+^) of these two CPAP-E1235V mutant clones (Figure 2C; Supplementary Figure S1), indicating that the mutant cells are more susceptible to apoptosis. In summary, our results indicate that both CPAP-WT and CPAP-E1235V mutant iPSCs exhibit similar pluripotency properties, but the mutant clones are more prone to apoptosis.

### CPAP-E1235V Mutant iPSCs Produce Short Centrioles but Abnormally Long Cilia

**FIGURE 3:**
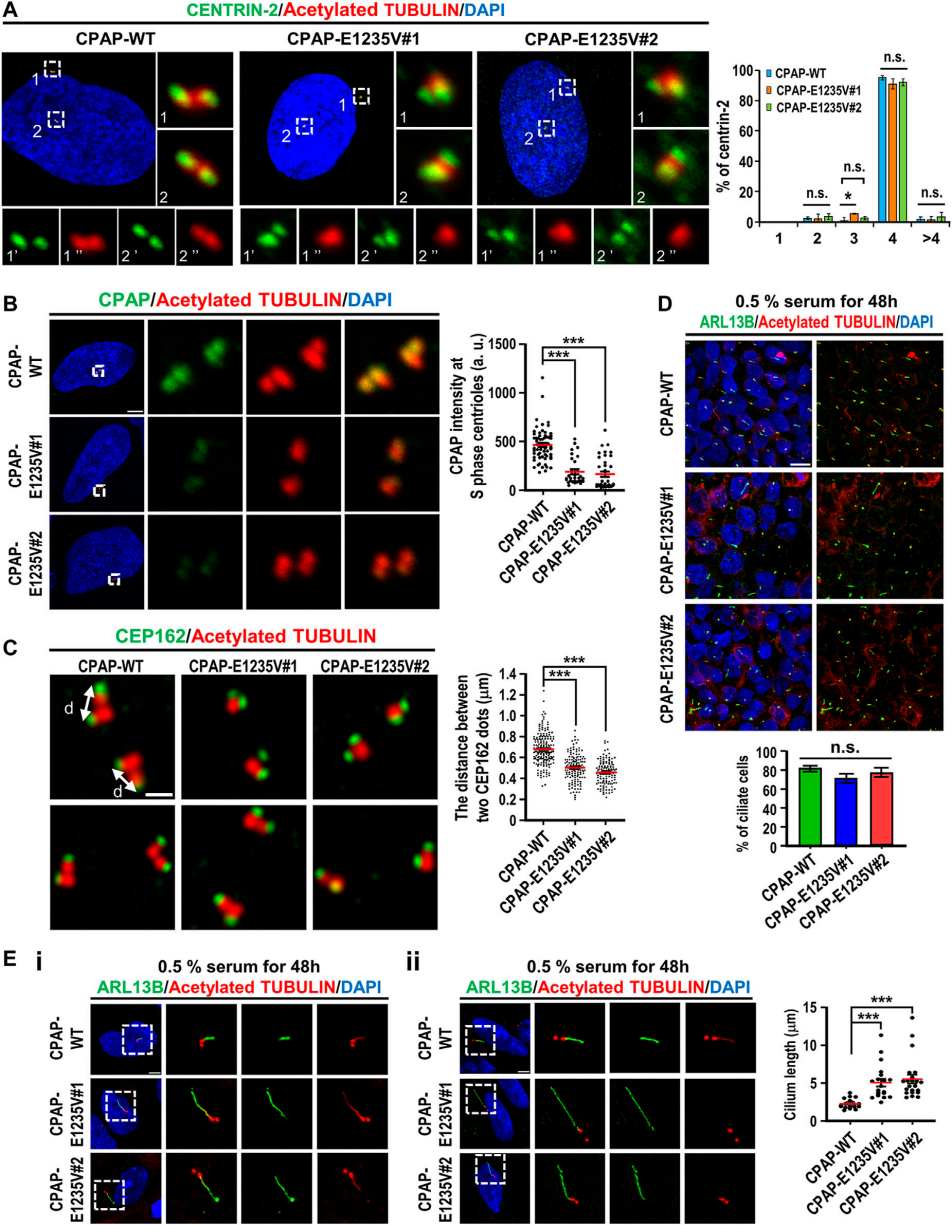
hiPSCs carrying CPAP-E1235V mutation displayed short centrioles and abnormally long cilia. Cells were synchronized at early S phase by treating aphidicolin (2 μg/ml) for 24 h (S phase) and released in fresh culture medium without aphidicolin for another 16 h (G2 phase). **(A)** Centriole numbers in control and two hiPSC mutant clones. Cells in the G2 phase were immunostained with antibodies against CENTRIN-2 (a centriole marker) and Acetylated-TUBULIN (Ac-TUB) (left) and the number of centriole was quantified (right) from three independent experiments. **(B)** Immunostaining and quantification of CPAP signals in S phase centrioles. *n = 61* for CPAP-WT; *n = 30* for CPAP-E1235V#1; *n = 37* for CPAP-E1235V#2. **(C)** Airyscan images of centriole lengths by measuring the distance between two CEP162 dots in each pair of centrioles. *n = 192* for CPAP-WT; *n = 134* for CPAP-E1235V#1; *n = 122* for CPAP-E1235V#2. **(D,E)** Immunostaining of CPAP-WT and mutant cells with antibodies against ARL13B (a ciliary membrane marker) and Ac-TUB (a centriole and ciliary axoneme marker). The cells were treated with 0.5% low serum starvation for 48 h. **(D)** The number of ciliated cells. *n = 265* for CPAP-WT; *n = 95* for CPAP-E1235V#1; *n = 196* for CPAP-E1235V#2. Data are presented as the mean ±SEM from a pool of n cells from three independent experiments. **(E)** Quantification of ciliary length. *n = 20* for CPAP-WT; *n = 20* for CPAP-E1235V#1; *n = 22* for CPAP-E1235V#2. Two types of long cilia were observed in the mutant cells. One type of long cilia displays long ciliary membranes (ARL13B^+^) and long ciliary axonemes (Ac-TUB^+^) **(Ei)**, while the other type of cilia exhibit long ciliary membranes (ARL13B^+^) but short axonemes (Ac-TUB^+^) **(Eii)**. Data are presented as the mean ±SEM. ***p < 0.001. Scale bar: 5 μm in **(A,B,E)**, 0.5 μm in **(C)**, 10 μm in **(D)**.

Our team and others researchers previously reported that CPAP plays a key role in the process of centriole elongation (Kohlmaier et al., 2009; Schmidt et al., 2009; Tang et al., 2009), and that the CPAP-E1235V mutant exhibits low binding activity to STIL, a key regulator of centriole biogenesis (Tang et al., 2011). To examine whether the CPAP-E1235V mutant affects centriole biogenesis, we first examined the centriole numbers in hiPSC-derived CPAP-WT and CPAP-E1235V mutant clones in the G2 phase and found that both CPAP-WT and the mutant clones contained similar centriole numbers (Figure 3A). Interestingly, the signal intensity of the CPAP-E1235V protein on the S-phase centrioles was significantly reduced in two CPAP-E1235V mutant cells (Figure 3B). To examine whether the CPAP-E1235V mutation affects centriole elongation, the cells were synchronized in the G2 phase and stained with antibodies against CEP162 (a distal-end marker) and acetyl TUBULIN (a marker of centriole) as previously described (Chang et al., 2016). The G2-phase centrioles were chosen because the duplicated centrioles could be easily distinguished from one another and the procentriole was at nearly its full length. We found that the mean distance between two CEP162 dots in the CPAP-E1235V#1 (∼0.5 μm) and CPAP-E1235V#2 (∼0.45 μm) cells was significantly shorter than that in the CPAP-WT (∼0.67 μm) cells (Figure 3C). A further analysis revealed that the cilia number was not altered in the E1235V mutant iPSCs (Figure 3D); however, they did produce abnormally long cilia (Figure 3E). Interestingly, two types of abnormally long cilia were observed. One population of mutant cells exhibit long cilia (∼10% in both CPAP-E1235V#1 and CPAP-E1235V#2 cells), which display long ciliary membranes (positive for ARL13B, which is a ciliary membrane marker) and long ciliary axonemes (positive for acetyl TUBULIN, which is a ciliary axoneme marker) (Figure 3Ei). In contrast, the majority of long cilia in mutant cells (>90%) exhibit long ciliary membranes (ARL13B^+^) with short axonemes (acetyl TUBULIN^+^), in which the acetyl TUBULIN signals did not appear to elongate with the corresponding ciliary membrane (Figure 3Eii); the reason for this is not known.

**FIGURE 4:**
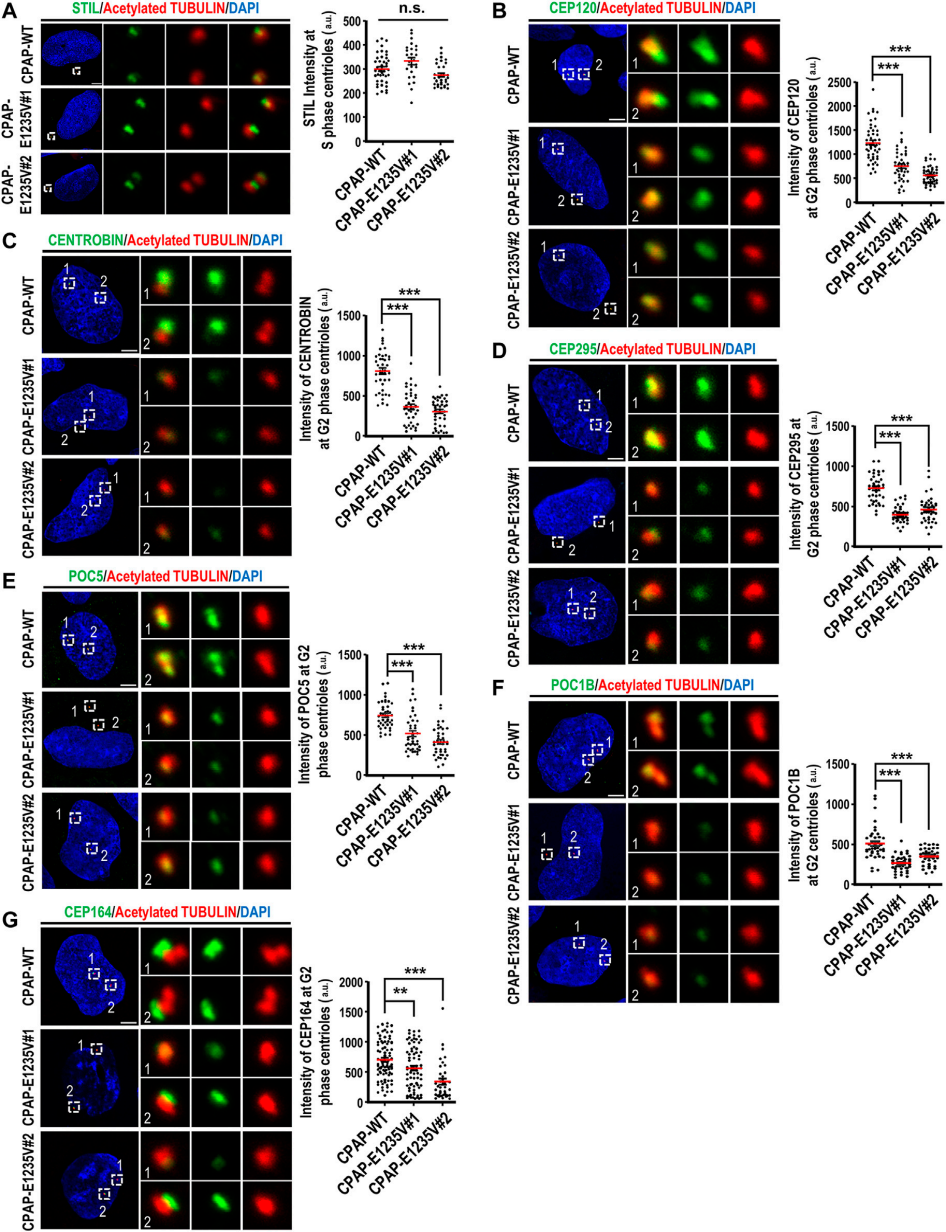
CPAP-E1235V mutation impaired the recruitment of centriole elongation proteins onto the centrioles. Cells were synchronized in the early S phase by treatment with aphidicolin (2 μg/ml) for 24 h. They were then fixed immediately with methanol (S phase centrioles) or released into fresh culture medium without aphidicolin for another 16 h (G2 phase centrioles). CPAP-WT or mutant cells in the S phase or G2 phase were immunostained with antibodies against the following centriolar proteins. **(A)** STIL, *n = 42* for CPAP-WT; *n = 25* for CPAP-E1235V#1; *n = 34* for CPAP-E1235V#2. **(B)** CEP120, *n = 50* for CPAP-WT; *n = 42* for CPAP-E1235V#1; *n = 48* for CPAP-E1235V#2. **(C)** CENTROBIN, *n = 40* for all groups. **(D)** CEP295, *n = 40* for all groups. **(E)** POC5, *n = 42* for CPAP-WT; *n = 40* for CPAP-E1235V#1; *n = 40* for CPAP-E1235V#2**. (F)** POC1B, *n = 40* for all groups. **(G)** CEP164, *n = 88* for CPAP-WT; *n = 74* for CPAP-E1235V#1; *n = 38* for CPAP-E1235V#2. All data are presented as mean ±SEM from a pool of n cells from three independent experiments. n.s: not significant; **p < 0.01; ***p < 0.001. Scale bar: 5 μm.

Since the CPAP-E1235V mutation led to the production of shortened centrioles in hiPSCs, we next examined how the CPAP-E1235V mutation affects the localization of several known centriolar proteins on the G2 centrioles, including an early-born centriolar protein STIL, centriole elongation proteins (CEP120, CENTROBIN, and CEP295), later-born centriolar proteins (POC1B and POC5), and a distal appendage protein CEP164. Our results showed that the CPAP-E1235V mutation had no apparent effect on the recruitment of the early-born centriolar protein STIL (Figure 4A), an upstream effector of CPAP (Tang et al., 2011), to the mutant cells. However, it did affect the recruitment of several reported centriolar elongation proteins (CEP120, CENTROBIN, and CEP295) (Figures 4B–D), the later-born proteins (POC5 and POC1B) (Figures 4E,F), and the distal appendage protein CEP164 (Figure 4G), to the centrioles. Together, our results indicate that the CPAP-E1235V mutation produces short centrioles with a defect in the recruitment of the centriolar proteins (CEP120, CENTROBIN, and CEP295) involved in procentriole elongation, the later-born proteins (POC1B and POC5) for building the distal-half centrioles, and the distal appendage protein CEP164.

### Human-Induced Pluripotent Stem Cell-Derived Organoids Phenocopy Many Morphological Features During Early Human Cerebral Development

**FIGURE 5:**
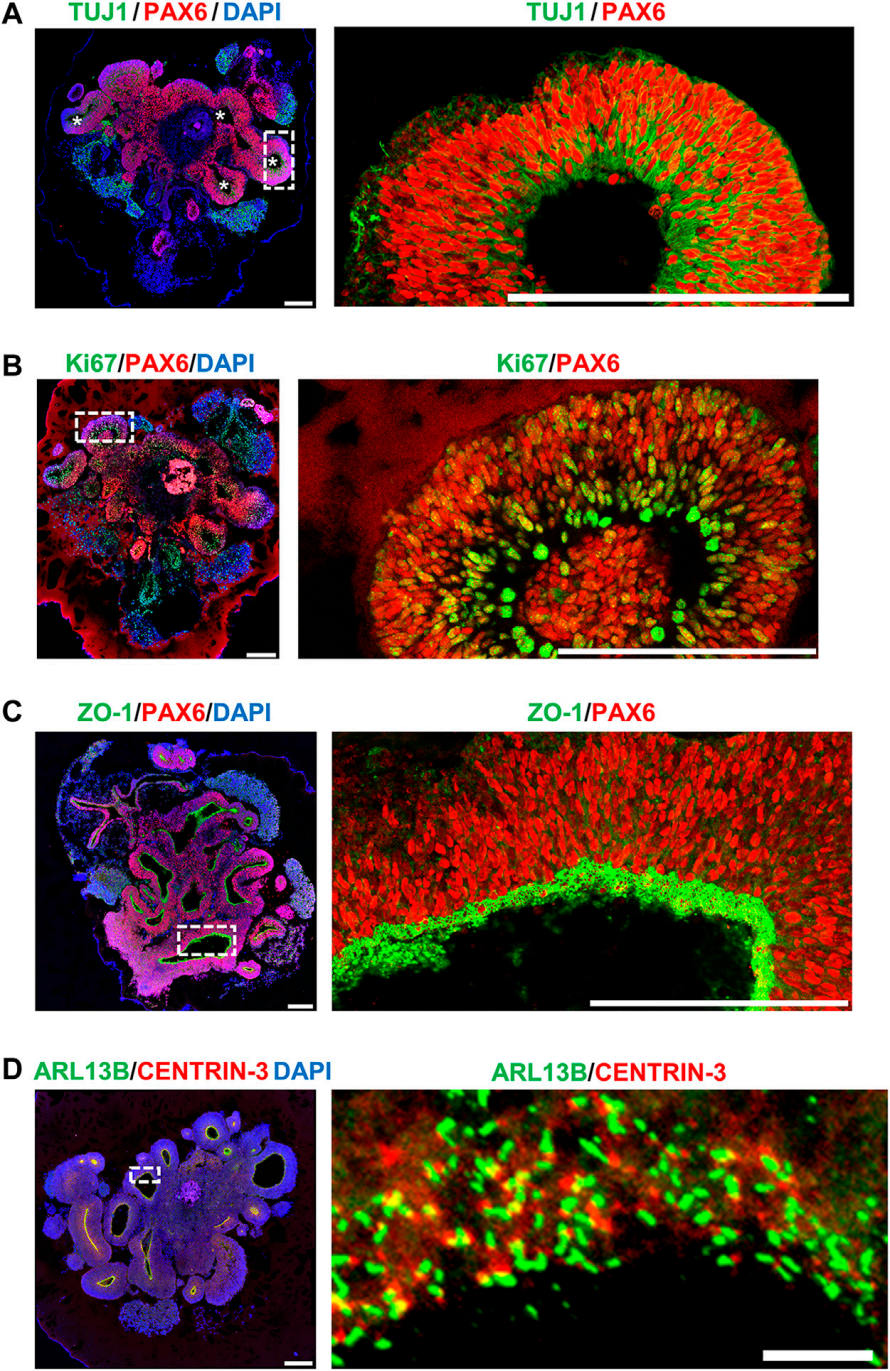
Characterization of hiPSC-derived brain organoid at Day 27 after culture. Immunostaining of the cryosections obtained from d27 organoids for **(A)** TUJ1 (a neuron marker)/PAX6 (a NPC marker), **(B)** Ki67 (a cell proliferating marker)/PAX6, **(C)** ZO-1 (a tight junction marker)/PAX6, and **(D)** ARL13B (a ciliary membrane marker)/CENTRIN-3 (a centriole marker). The cilia protruded from the apical surface in 27-day-old brain organoids. Scale bar: 200 μm in **(A–D)**, 10 μm in enlarge **(D)** (right). White stars in **(A)** indicate the ventricle-like cavities in the d27 organoids. The enlarged image in [**(A)**, right] is a 90-degree left turn from the left image.

To model early human cerebral development, we adopted an established protocol for the hiPSC-derived brain organoid culture system described by Lancaster and Knoblich (2014). Our results showed that at Day 27 after culture, the hiPSC-derived brain organoid generated ventricle-like cavities (Figure 5A, left panel) surrounded by radially organized cells, which were positively stained for the radial glia cell (RGC) marker PAX6 (red, Figure 5A). The marker for cell proliferation Ki67 (green, Figure 5B) was also detected at the neural progenitors close to the ventricle. Further analysis showed that the apical progenitor cell layer of the ventricle-like cavities in the 27d-old organoids positively stained for ZO-1 (an apical junction protein marker, green, Figure 5C), and the ARL13B-labelled cilia (green) were protruded from the surface of the apical RGCs (Figure 5D), which shows a pattern resembling the apical side of the ventricular zone (VZ) of the developing cerebral cortex.

**FIGURE 6:**
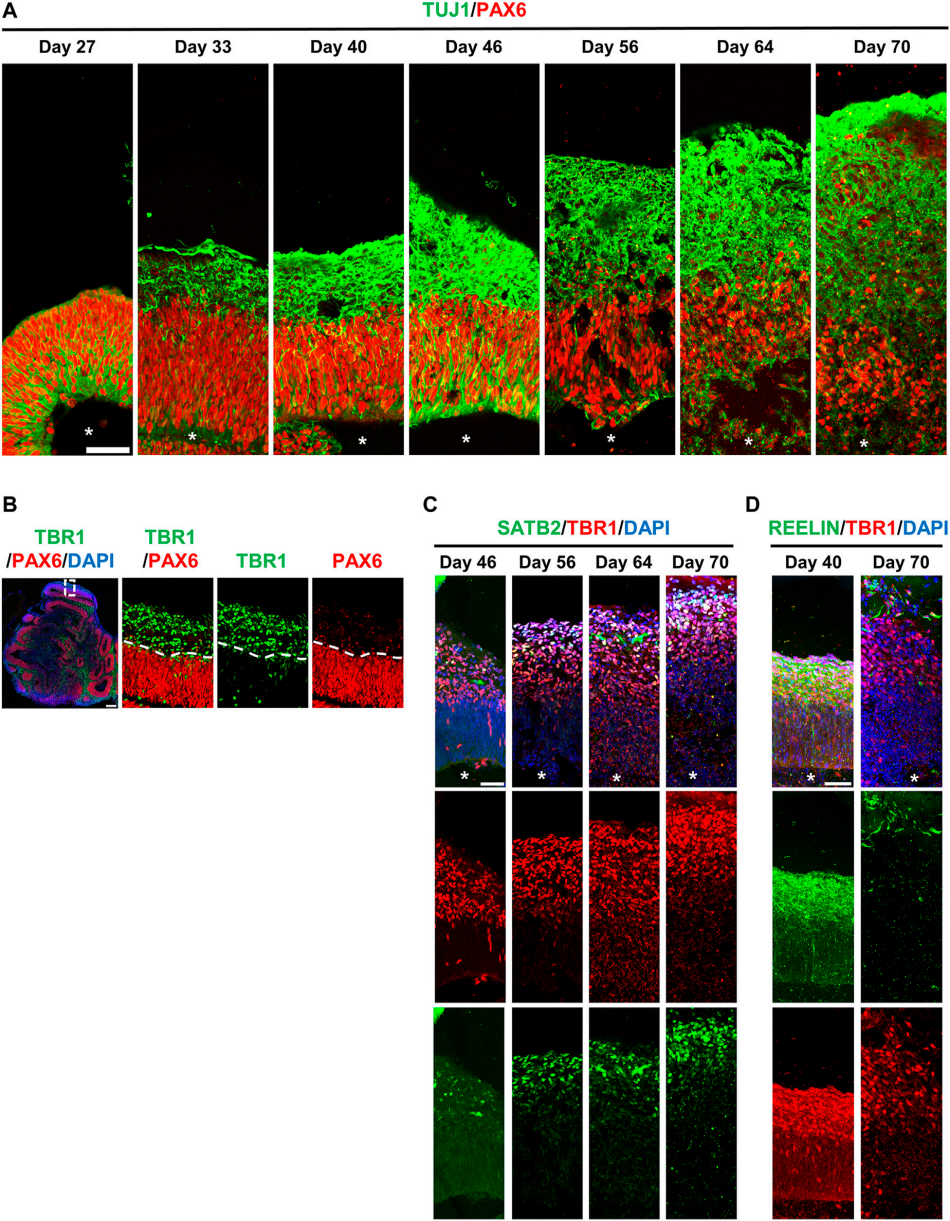
hiPSCs-derived brain organoids recapitulate many cellular features of the developing human brain. **(A)** Neurogenesis in developing brain organoids. Immunostaining of the cryosection of developing organoids at the indicated time points for TUJ1 (a neuron marker)/PAX6 (a NPC marker). **(B)** Immunostaining of the 46-day-old brain organoid with antibodies against TBR1 (an early-born neural marker)/PAX6. (**C,D)** Immunostaining of developing organoids at the indicated time points for SATB2 (a later-born neural marker)/TBR1 **(C)** and for REELIN (a Cajal–Retzius cell marker)/TBR1 **(D)**. White stars: lateral ventricle-like cavities. Scale bar: 50 μm in **(A,C,D)**; 200 μm in **(B)**.

To assess the neurogenesis in hiPSC-derived brain organoids, we traced PAX6-labelled RGCs and TUJ1-labelled differentiated neurons at different time points during the progression of brain organoids. At Day 27 after culture, we predominantly observed PAX6-labelled RGCs (red, Figure 6A) and a small portion of TUJ1-positive cells (green, Figure 6A) located radially at the ventricular-like (VZ) zone. However, these TUJ1-labelled cells moved up to the putative cortical plate (CP), which gradually increased in thickness by increasing the number of TUJ1-positive cells during organoid development (from 27d to 70d) (Figure 6A). Unexpectedly, a small number of TUJ1-positive neurons could be detected at the region close to the ventricle-like cavities in some brain organoids (Figures 5A, 6A), implying that some degree of misdifferentiation may be occurred in this culture system (Bhaduri et al., 2020). Nevertheless, these TUJ1 positive signals were not detected at the primitive CP in the 27d-old organoids (Figures 5A, 6A). Further analysis showed that the early-born TBR1-labelled differentiated neurons were located outside of PAX6-labelled neural progenitor cell layer (d46) during brain organoid development (Figure 6B).

Similar to previous reports (Li et al., 2017), these early-born TBR1^+^ neurons were produced earlier than the later-born SATB2^+^ neurons during corticogenesis, which led to the SATB2^+^ neurons being localized more superficially than the TBR1^+^ neurons (Figure 6C), suggesting that the lamination pattern is similar to that of the developing human neocortex. In addition, reelin-positive cells were detected along the basal surface on 40d-organoids and gradually decreased on Day 70 (Figure 6D). This suggests the presence of Cajal–Retzius (CR)-like neurons at the surface of the developing cerebral cortex, which were later eliminated through cell death in the early postnatal stages: a temporal patterning similar to that of the developing human brain (Renner et al., 2017). Together, our hiPSC-derived brain organoids recapitulated many cerebral phenotypical features seen in the developing human brain.

### CPAP E1235V Mutation Produces Small Organoids *via* p53-dependent Neuronal Cell Death, Induces NPC Spindle Misorientation, and Triggers Premature Neuronal Differentiation

**FIGURE 7:**
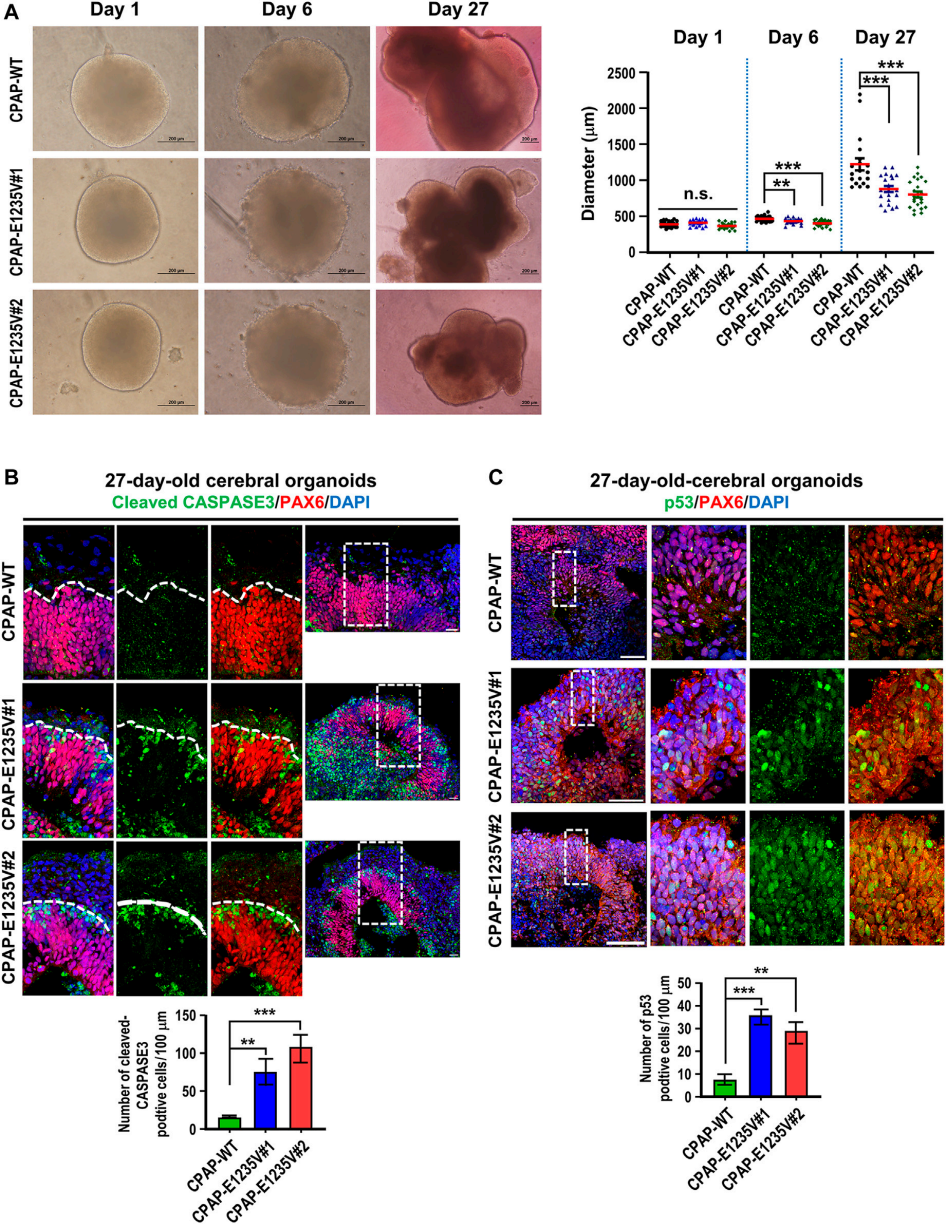
CPAP-E1235V mutant organoids revealed the microcephaly phenotype. **(A)** Bright-filed morphology of CPAP-WT and mutant brain organoids at the embryoid body stage (Day 1 and Day 6) and differentiation stage (Day 27). The pool of larger brain organoids from WT and mutant groups was quantified and shown in right. *n= 19* for CPAP-WT; *n = 21* for CPAP-E1235V#1; *n= 22* for CPAP-E1235V#2. Data are presented as mean ±SEM. **(B)** Immunostaining and quantitation of cleaved CASPASE 3 positive cells in 27d-old WT and mutant brain organoids. *n = 5* for CPAP-WT; *n = 4* for CPAP-E1235V#1; *n = 4* for CPAP-E1235V#2. All data are presented as mean ±SEM from three independent experiments. **(C)** Immunostaining and quantification of p53 positive cells in 27-day-old WT and mutant brain organoids. *n = 4* for CPAP-WT; *n = 3* for CPAP-E1235V#1; *n = 3* for CPAP-E1235V#2. All data are presented as mean ±SEM from three independent experiments. n. s: not significant; **p < 0.01; ***p < 0.001. Scale bar: 200 μm in **(A)**, 20 μm in **(B),** 50 μm in **(C)**.

The CPAP-E1235V mutation was previously reported to cause MCPH in human patients (Bond et al., 2005). To investigate how the CPAP-E1235V mutation causes MCPH, we examined the phenotypical and pathological features in wild-type and mutant organoids. We first seeded an equal number of hiPSCs carrying the WT or CPAP-E1235V mutation (9000 cells at the starting point) to produce embryoid bodies (EBs). All CPAP-E1235V mutant clones (#1, #2) and CPAP-WT, exhibited similar sized EBs at Day 1 after culture (Figure 7A). However, at Day 6 and Day 27 after culture, the CPAP-E1235V mutant clones gradually produced smaller organoids as compared to the isogenic wild-type control (Figure 7A). To investigate the cause of the size reduction in CPAP-E1235V mutant organoids, we immunostained the 27-day organoids with antibodies against cleaved CASPASE 3 (an apoptosis marker) and p53. Our results showed that the CASPASE 3-activated cells were significantly increased (Figure 7B). This was accompanied with an increase in p53 nuclear accumulation at the PAX6-labeled RGCs in the mutant organoids (Figure 7C). Interestingly, the apoptotic cells seem to be mainly restricted in PAX6-negative cells, which are located just above the PAX6-positive cell zone (Figure 7B; enlarged images in Supplementary Figure S2), while the increased p53 nuclear signal was majorly detected in PAX6-positive progenitors (Figure 7C; enlarged images in Supplementary Figure S3). Although, some CASPASE 3-positive cells were also observed in the lumen of the ventricle-like cavities in some mutant and wild-type organoids, these apoptotic cells are PAX6 negative, which may be generated due to environment-induced cellular stress in the organoid culture system (Bhaduri et al., 2020). Together, these findings suggest that the CPAP-E1235V mutation induced neuronal cell death *via* a p53-dependent manner.

**FIGURE 8:**
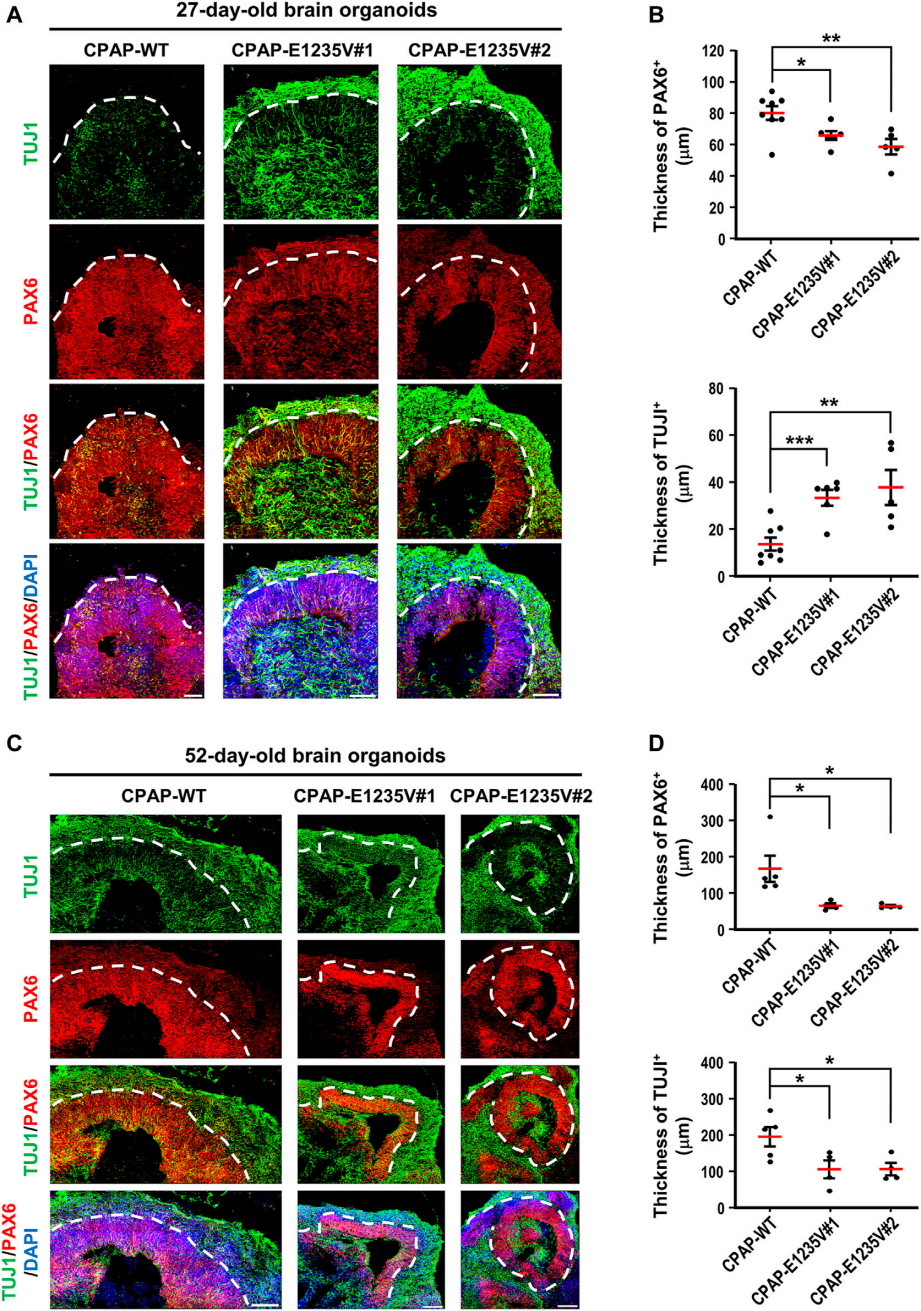
CPAP-E1235V-derived brain organoid exhibited premature neuronal differentiation. **(A)** Immunostaining of TUJ1-positive neurons and PAX6-positive NPCs in 27-day-old WT and mutant brain organoids. The thickness of TUJ1-positive and PAX6-positive cell layers in 27-day-old WT and mutant organoids were quantified and are shown in **(B).** *n = 8* for CPAP-WT; *n = 6* for CPAP-E1235V#1; *n = 5* for CPAP-E1235V#2. **(C,D)** Immunostaining of TUJ1-positive neurons and PAX6-positive NPCs **(C)** and their corresponding cell layer thickness in 52-day-old WT and mutant brain organoids were quantified and are shown in **(D)**. *n = 5* for CPAP-WT; *n = 4* for CPAP-E1235V#1; *n = 4* for CPAP-E1235V#2. All data are presented as mean ±SEM. *p < 0.05; **p < 0.01; ***p < 0.001. Scale bar: 50 μm in **(A)**, 100 μm in **(C)**.

**FIGURE 9:**
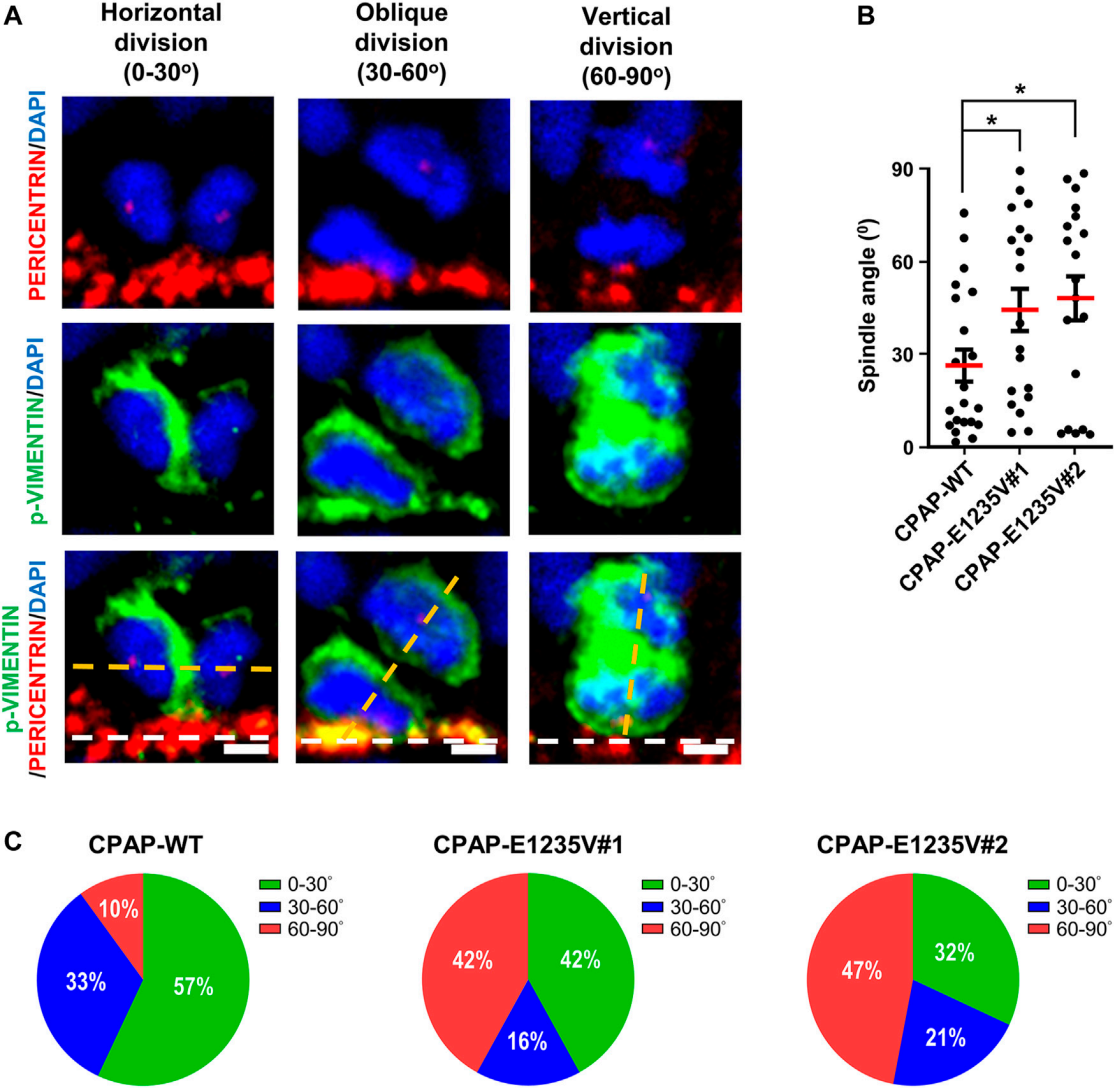
CPAP-E1235V mutation induces NPC spindle misorientation in mutant brain organoids. **(A)** Representative images of NPC division in 27-day-old WT and mutant brain organoids with horizontal, oblique, and vertical mitotic spindle orientations at the ventricular surface (white lines). The spindle angle of mitotic anaphase NPCs (labeled by p-VIMENTIN) was calculated using a line (yellow) connecting two centrosomes (labelled by PERICENTRIN) and another line (white) representing the apical surface using the Image J software. Part of the PERICENTRIN-labelled centrosomes in NPCs is not visible due to the image being obtained from a single Z-stack (0.8 μm thickness), but not the merged Z-stacks. **(B,C)** Quantification of the spindle angle of the mitotic anaphase NPCs in 27-day-old CPAP-WT and mutant organoids. CPAP-WT NPCs divided horizontally in the majority of cases (0–30° angle), whereas mutant NPCs displayed many vertical orientations **(C)**. Results were obtained from a pool of NPCs (n) from at least three independently collected organoids in each group. *n = 21* NPCs for CPAP-WT; *n = 19* NPCs for CPAP-E1235V#1; *n = 19* NPCs for CPAP-E1235V#2 **(C)**. Data are presented as mean ±SEM. ***p < 0.001. Scale bar: 10 μm.

We next measured the thickness of the VZ-like and CP-like region of the 27d-brain organoids by immunostaining for PAX6 (a neural progenitor maker) and TUJ1 (a neuronal marker). Our results showed that the thickness of the PAX6-labeled VZ-like layer decreased (Figures 8A,B, right upper panel) and the TUJ1-labeled CP-like layer increased (Figures 8A,B, right bottom panel) in the mutant organoids as compared to the wild-type control, suggesting a reduction in neuronal progenitors at the VZ and an increase in differentiated TUJ1^+^ neurons in the 27-day mutant organoids. However, at Day 52, the thickness of both the PAX6- (Figures 8C,D, right upper panel) and TUJ1-labeled layers (Figures 8C,Dright bottom panel) were significantly reduced in the mutant organoids. This may have been due to neuronal cell death, which occurred previously (d27), resulting in a reduction in the differentiated neuronal layers in later developmental stages (d52). Further analysis of the spindle orientation of radial glia cells (Figure 9A) at VZ (vRGs) using p-VIMENTIN (a mitotic division marker) and PERICENTRIN (a centrosome marker) staining showed that control vRGs preferentially divided horizontally (0–30° angle, ∼57%), while mutant vRGs displayed many vertical orientations (Figures 9B,C), suggesting that the CPAP-E1235V mutation causes abnormal mitotic spindle orientation in vRGs. Taken together, our results suggest that the CPAP-E1235V mutation produces small organoids *via* p53-dependent neuronal cell death, induces vRG spindle misorientation, and triggers premature neuronal differentiation.

## Discussion

Our recent studies showed that the microcephaly genes, which encode centriolar proteins (CPAP, STIL, CEP135, and RTTN), play an important role in the early stage of centriole duplication (Tang et al., 2009; Tang et al., 2011; Lin et al., 2013a; Chen et al., 2017; Tsai et al., 2019). Using the CRISPR/Cas9 genome editing system, we produced two hiPSC lines carrying a homozygous *CPAP/CENPJ* missense mutation (c.3704A > T; E1235V). Interestingly, the CPAP-E1235V mutant hiPSCs produced short centrioles (Figure 3C) but abnormally long cilia (Figure 3E). A similar finding that vRGs possess long cilia was also observed in CPAP-E1235V mutant organoids (Supplementary Figure S4). It was reported that the CPAP-E1235V mutant reduces its binding to the microcephaly protein STIL and inhibits centriole duplication (Tang et al., 2011). Since STIL functions upstream of CPAP (Tang et al., 2011), we here found that CPAP-E1235V does not affect STIL targeting to the procentrioles (Figure 4A), but the recruitment of CPAP-E1235V to the STIL-containing centrioles is reduced (Figure 3B). Thus, a possible scenario of producing short centrioles is that a reduced amount of CPAP-E1235V present on the procentriole during the early stage of centriole duplication impairs the recruitment of several centriolar elongation proteins (CEP120, CENTROBIN, and CEP295) (Figures 4B–D) and the later-born distal-half proteins (POC1B and POC5) to the centrioles (Figures 4E,F), which results in the inhibition of full-length centriole assembly.

Interestingly, two populations of long cilia were observed in the mutant hiPSC cells. One population displayed long ciliary membranes (ARL13B^+^) and long ciliary axonemes (acetyl TUBULIN^+^) (Figure 3Ei), while the other population exhibited long ciliary membranes (ARL13B^+^) but short axonemes (acetyl TUBULIN^+^) (Figure 3Eii). Recently, Gabriel et al. (2016) reported that Seckel patient-derived fibroblasts that carried a splice-site mutation in *CPAP* produced long cilia and displayed retarded cilium disassembly. They proposed that CPAP provides a scaffold for the recruitment of the cilium disassembly complex, including Nde1, Aurora A, and OFD1, to the ciliary base for timely cilium disassembly (Gabriel et al., 2016). Currently, it is not clear whether the CPAP-E1235V mutant uses a similar mechanism or another mechanism to regulate its cilia length. Furthermore, previous studies showed that CPAP plays roles in regulating centriole elongation, PCM recruitment, and spindle position (Kohlmaier et al., 2009; Schmidt et al., 2009; Tang et al., 2009; Kitagawa et al., 2011; Zheng et al., 2014), and the molecular basis for CPAP-tubulin interaction in controlling centriolar and ciliary length was proposed (Zheng et al., 2016). Future analyses including Cryo-EM and X-ray crystallography are required to elucidate the underlining mechanism.

Genetic microcephaly syndromes in humans are relatively rare and genetic mouse models cannot fully phenocopy the pathological aspects of human MCPH. By studying CPAP-E1235V mutant brain organoids, we provided novel insights into the development of human MCPH. First, the CPAP-E1235V mutation produces small brain organoids as compared to CPAP-WT organoids (Figure 7A), possibly by reducing the amount of NPCs and their derived neurons *via* p53-dependent apoptosis (Figures 7B,C) and premature neuronal differentiation. Mitotic catastrophe was delineated as a type of cell death resulting from aberrant mitosis (Kroemer et al., 2009) and three different modes have been described: 1) Immediate mitotic death (cells die without exiting mitosis), 2) subsequent G1 cell death (cells reach the G1 phase of the subsequent cell cycle and undergo cell death), and 3) senescence (cells exit mitosis and undergo senescence) (Vitale et al., 2011). Our previous study showed that complete loss of Cpap induces mitotic abnormalities in neural progenitors and subsequent p53-mediated apoptosis in cells located outside the ventricular zone in *Cpap* knockout mice (Lin et al., 2020). We hypothesized that centriole loss due to *Cpap* gene deletion may induce a mitotic catastrophe in neural progenitor that drives neuronal cell death *via* a G1 cell death mode (Lin et al., 2020). Consistent with this finding, our present results further elucidated that the apoptosis mainly occurred in PAX6-negative cells, which are located just above the PAX6-positive cell zone (Figure 7B; enlarged images in Supplementary Figure S2). Interestingly, increased nuclear p53 signals were majorly detected in PAX6-positive progenitors (Figure 7C; enlarged images in Supplementary Figure S3). Together, our results support a concept that centriole/centrosome defects caused by complete loss of Cpap or CPAP-E1235V mutation in neural progenitors may induce an increase of p53 accumulation in the nucleus of PAX6-positive progenitors (Supplemental Figure S3), followed by triggering a p53-mediated apoptosis in subsequent G1 cell death in PAX6-negative cells. The neuronal cell death was also observed in several mouse microcephaly models (Insolera et al., 2014; Marjanović et al., 2015; Little and Dwyer, 2018; Lin et al., 2020) and in hiPSC-derived brain organoid carrying microcephaly *WDR62* mutation (Zhang et al., 2019), which are consistent with our findings. Recently, Phan et al. reported that centrosome defects cause neuronal cell death by activating the 53BP1-USP28-TP53 mitotic surveillance pathway (Phan et al., 2021) in a mouse model with microcephaly. It will be interesting to investigate whether a similar mechanism is also present in CPAP-E1235V mutant brain organoids.

Furthermore, the CPAP-E1235V mutation can induce NPC spindle misorientation (Figure 9) and trigger premature neuronal differentiation (Figure 8A) in mutant organoids. Interestingly, we found that CPAP-E1235V mutation produced smaller organoids with increased the TUJ1-positive cell layer but decreased PAX6-positive cell layer (Figures 8A,B). It is possible that the spindle misorientation induced by CPAP-E1235V mutation affects the symmetric and asymmetric division of NPCs at the early stage of corticogenesis (27d-old organoid), resulting in premature neuron differentiation and, such an effect leads to an overall reduction in the NPC pool. However, the underlining mechanism is not clear. Previous studies identified several key pathways that regulate asymmetric/symmetric cell division in neural progenitor cells (NPCs) in fly and vertebrate models (Jan and January 2001; Wodarz and Huttner, 2003; Du and Macara, 2004; Knoblich, 2008). Among these, the partitioning-defective (PARD) protein complex and the evolutionary conserved NuMA/LGN/G*α*I complex are of particular interest. One of the main functions of the PARD3 and NuMA complexes is involved in asymmetric cell division and spindle orientation (Jan and January 2001; Du and Macara, 2004). We previously showed that loss of Cpap in neural progenitors in microcephalic mice perturbs the localization of PARD3 and N-cadherin to the apical membrane surface (apical end-foot), which impairs the junctional integrity at the ventricular surface. Such a defect could induce premature neuronal differentiation, leading to massive heterotopia involving CUX1^+^ upper-layer differentiated neurons (Lin et al., 2020). We further demonstrated that loss of the microcephaly protein RTTN induced abnormal localization of cortical NuMA and perturbed the spindle position in cultured cells (Chou and Tang, 2021). Thus, it is possible that the PARD3 and/or the NuMA complexes may participate in asymmetric division and premature neuronal differentiation in the developing brain. In future studies, it will be interesting to investigate the functional roles of the PARD3 and the NuMA complexes in CPAP-E1235V-derived mutant organoids.

Finally, the results from our previous *Cpap* conditional knockout mice (Lin et al., 2020) and hiPSC-E1235V-derived mutant brain organoids (this report) provide possible centrosomal mechanisms that illustrates how a microcephalic brain is generated: 1) The centriole/centrosome defects caused by complete *Cpap* loss or CPAP-E1235V mutation may induce a p53-dependent neuronal cell death possibly *via* the G1 cell death mode; 2) the centriole/centrosome defects in NPCs perturb spindle orientation that affect symmetric/asymmetric division, resulting in premature neuronal differentiation and, as a result leading to an overall reduction in the NPC pool. Recently, Bhaduri et al. (2020) reported that cellular stress, such as endoplasmic reticulum (ER)- and/or glycolysis-activated stress, impair molecular subtype specification in cortical organoids, implying that the present organoid culture system may not actually represent the true process of developing human cerebral cortex. Since, it is difficult to directly analyze the pathogenesis of MCPH using human brain samples, our present study in combination with the results from the *Cpap* conditional knockout mice (Lin et al., 2020) provided valuable information for understanding the pathogenic process of MCPH. Future studies using transplantation of hiPSC-derived organoids carrying CPAP-E1235V mutation into the immunodeficient mouse brain should provide more in-depth mechanistic information for MCPH.

In conclusion, using the CRISPR-Cas9 genome editing system, we successfully generated hiPSCs carrying a naturally occurring missense mutation in the human *CPAP/CENP* gene. The hiPSC-CPAP-E1235V-derived mutant brain organoids provide a useful human cerebral-like model that mimics MCPH patients. This can be used to study the related functional mechanisms and in future biological drug screenings.

## Data Availability

The original contributions presented in the study are included in the article/Supplementary Material, further inquiries can be directed to the corresponding authors.
