# Supplementary material for: Modeling Human Primary Microcephaly With hiPSC-Derived Brain Organoids Carrying CPAP-E1235V Disease-Associated Mutant Protein
Source: Front Cell Dev Biol. 2022 Mar 2;10:830432. doi: 10.3389/fcell.2022.830432 (PMC8924525; doi:10.3389/fcell.2022.830432)

Supplementary Material contains four supplementary figures

Supplementary Figure S1. Detection of apoptosis in CPAP-WT and CPAP mutant hiPSC cell clones by TUNEL assay. **(A-C)** Separated channels with TUNEL/DAPI staining of hiPSCs of CPAP-WT **(A)**, CPAP-E1235V#1 **(B)**, and CPAP-E1235V#2 **(C)** from Figure 2C. Boxed regions (yellow) are enlarged at the right panel. White arrowheads indicate TUNEL positive signals co-localized with whole or fragmented nuclei (apoptotic bodies) in **(A)** CPAP-WT, **(B)** CPAP-E1235V#1, and **(C)** CPAP-E1235V#2 cell clones. A few apoptotic nuclei were also detected in CPAP-WT control cells. A statistical analysis of TUNEL-positive cells between CPAP-WT and CPAP-E1235V was shown in Fig 2C (bottom). Scale bar: 20 µm.

Supplementary Figure S2. Detection of apoptotic cells in CPAP-WT and mutant organoids by immunostaining of cleaved CASPASE 3 and PAX6. The DNA is counterstained with DAPI. **(A)** Enlarged images from Figure 7B. **(B)** Separated and enlarged channels with cleaved CASPASE 3/PAX6/DAPI staining of CPAP-WT, CPAP-E1235V#1, and CPAP-E1235V#2 organoids (27-day-old) from A. White arrows indicate $PAX6^-$/cleaved CASPASE 3^+ cells. Yellow arrowheads indicate $PAX6^+$/cleaved CASPASE 3^+ cells. **(C)** Quantification of the percentage of total cleaved $CASPASE3^+$ cells, $PAX6^-$/cleaved $CASPASE3^+$ cells, and $PAX6^+$/cleaved $CASPASE3^+$ cells out of total $DPAI^+$ cells. *n=5* for CPAP-WT; *n=4* for CPAP-E1235V#1; *n=4* for CPAP-E1235V#2. All data are presented as mean ±SEM from three independent experiments. ***P*<0.01; ****P*<0.001. Scale bar: 20 µm.

Supplementary Figure S3. The pattern of p53 activation in PAX6 cells in CPAP-WT and mutant organoids by immunostaining of p53 and PAX6. The DNA is counterstained with DAPI. **(A)** Enlarged images from Figure 7C. **(B)** Separated and enlarged channels with p53/PAX6/DAPI staining of CPAP-WT, CPAP-E1235V#1, and CPAP-E1235V#2 organoids (27-day-old) from A. White arrows indicate $PAX6^-/p53^+$ cells. Yellow arrowheads indicate $PAX6^+/p53^+$ cells. **(C)** Quantification of the percentage of total $p53^+$ cells, $PAX6^-/p53^+$ cells, and $PAX6^+/p53^+$ cells out of total $DAPI^+$ cells. *n=4* for CPAP-WT; *n=3* for CPAP-E1235V#1; *n=3* for CPAP-E1235V#2. All data are presented as mean ±SEM from three independent experiments. Scale bar: 50 µm.

Supplementary Figure S4. Long cilia in CPAP-E1235V mutant brain organoids. **(A)** Immunostaining of CPAP-WT and mutant organoid sections (52-day-old) with ciliary membrane marker ARL13B (green) and DAPI (blue). **(B)** Quantification of cilium length in CPAP-WT (mean: 0.58 µm), CPAP-E1235V#1 (mean: 1.43 µm), CPAP-E1235V#2 (mean: 1.42 µm) brain organoids (52-day-old). Results were obtained from a pool of cells (n) from at least three independent brain organoids in each group. *n=89* for CPAP-WT; *n=75* for CPAP-E1235V#1; *n=62* for CPAP-E1235V#2. ****P*<0.001. Scale bar: 10 µm.

Supplementary Figure S1

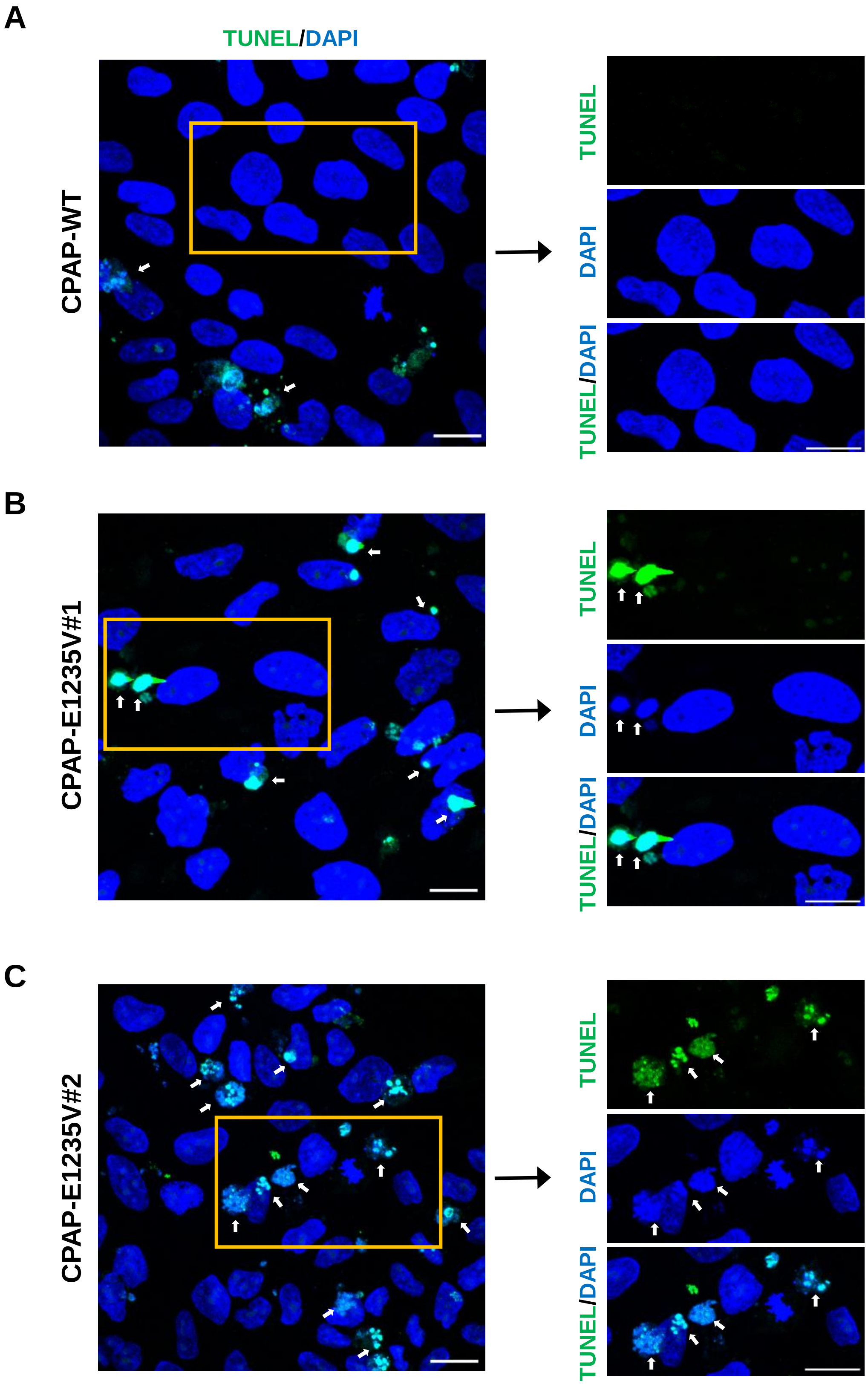

Supplementary Figure S2

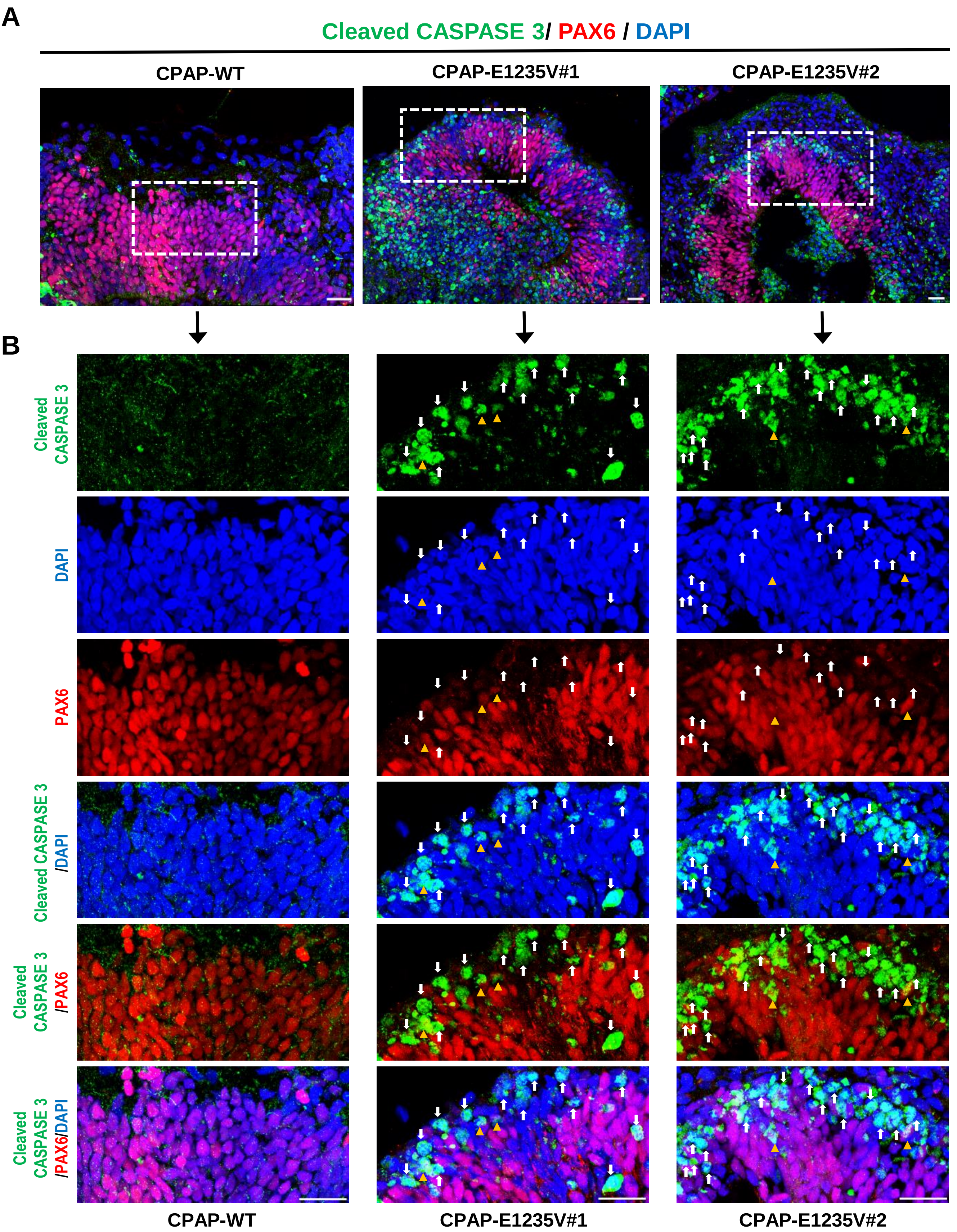

Supplementary Figure S2 (continued)

C

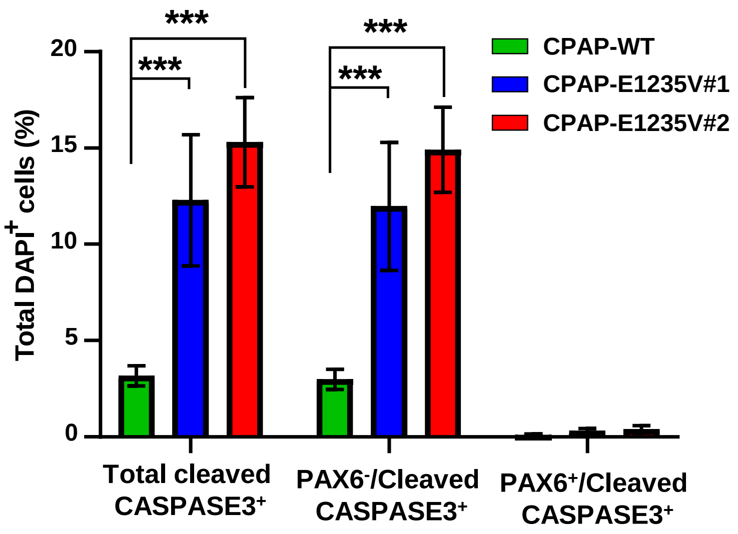

Supplementary Figure S3

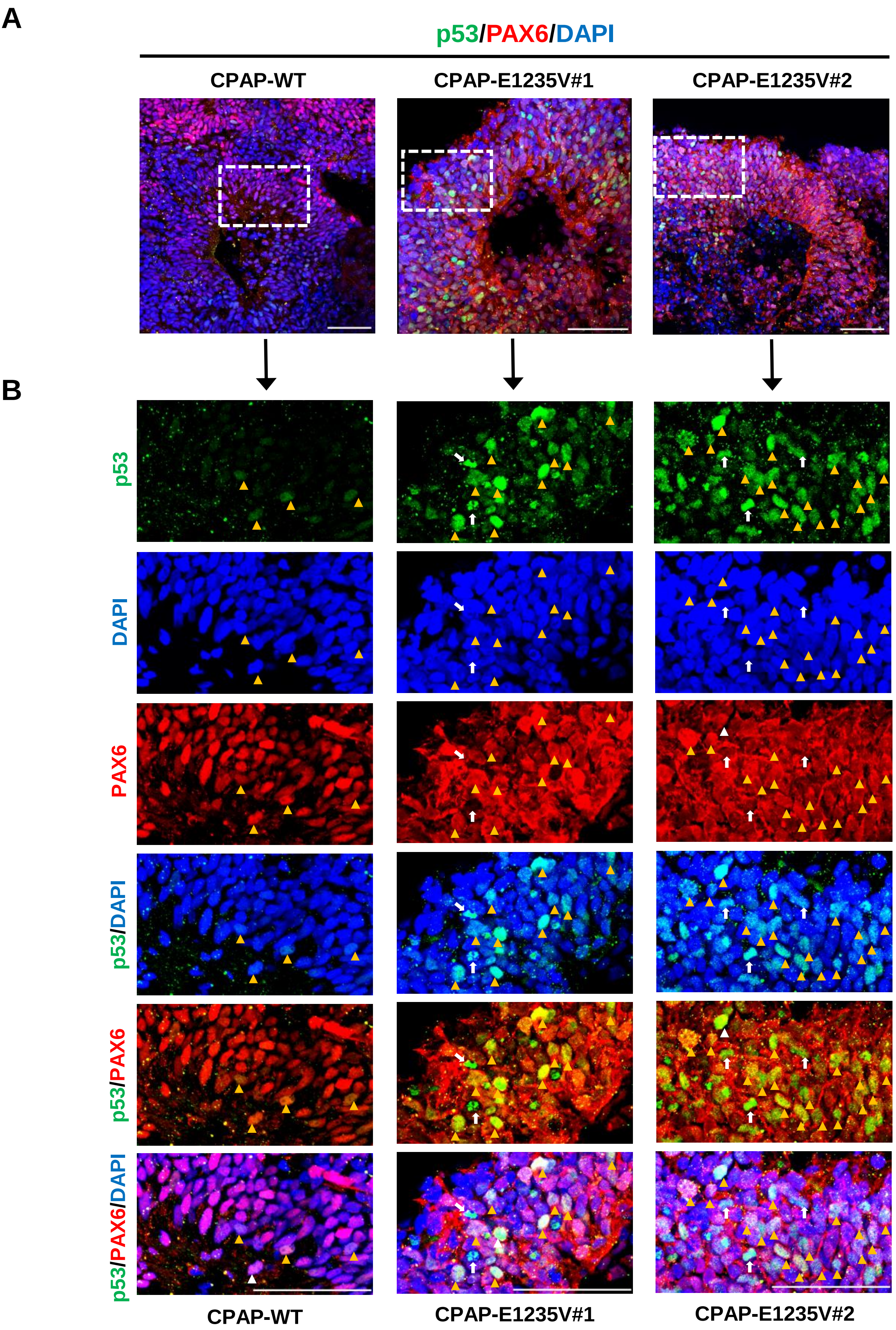

Supplementary Figure S3 (continued)

C

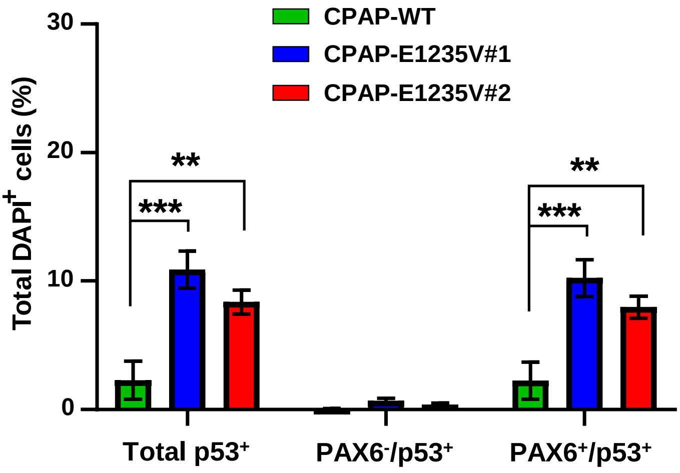

Supplementary Figure S4

A

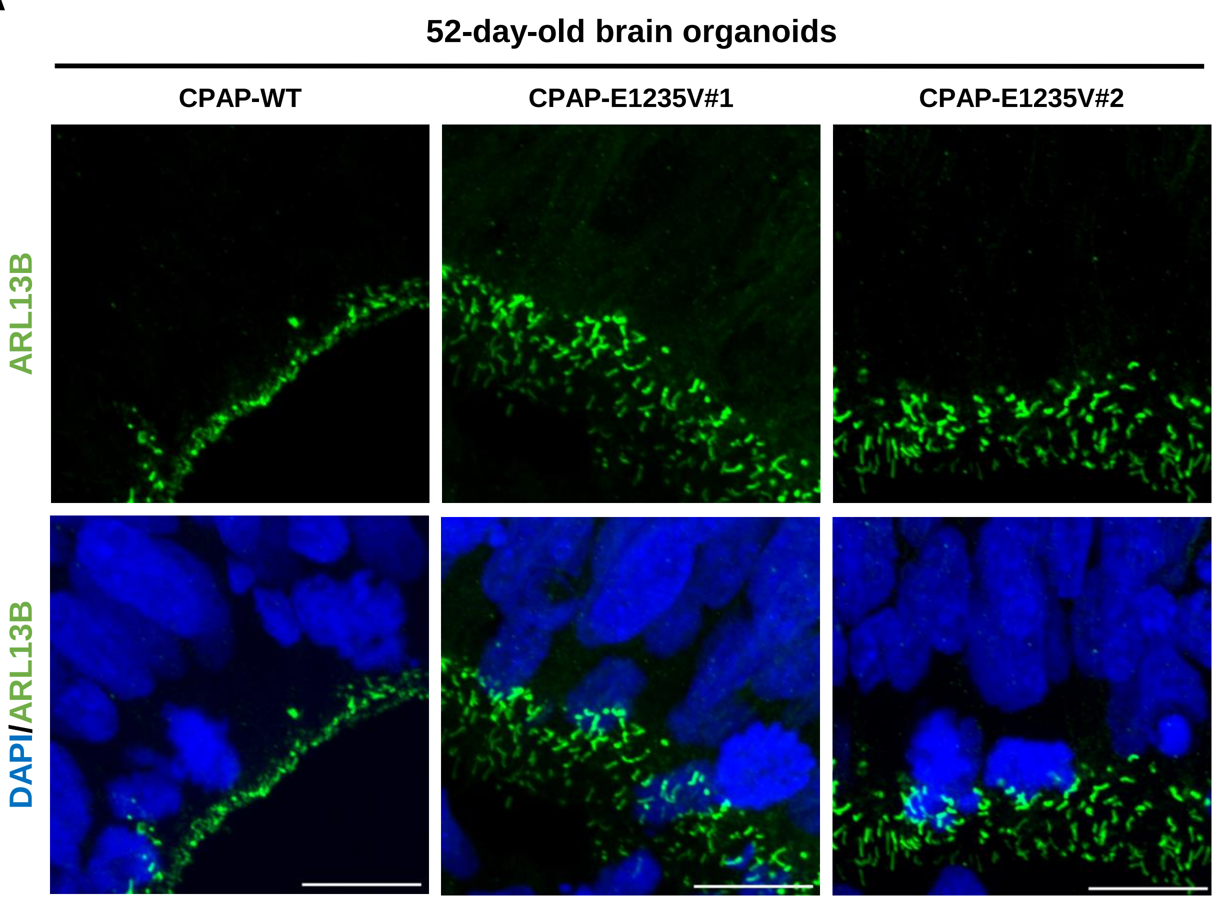

B

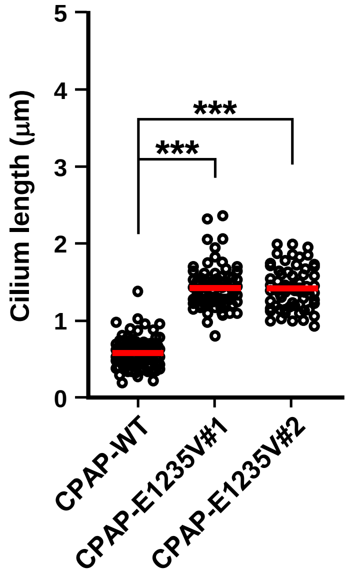

Supplement: Supplementary file 1 [file Image1.PDF]
